# Fighting Incivility in the Workplace for Women and for All Workers: The Challenge of Primary Prevention

**DOI:** 10.3389/fpsyg.2019.01805

**Published:** 2019-08-08

**Authors:** Annamaria Di Fabio, Mirko Duradoni

**Affiliations:** ^1^Department of Education, Languages, Interculture, Letters and Psychology (Psychology Section), University of Florence, Florence, Italy; ^2^Department of Information Engineering, University of Florence, Florence, Italy

**Keywords:** workplace incivility, primary prevention, workplace relational civility, positive relational management, emotional intelligence, well-being

## Abstract

This article discusses the role of several constructs, such as workplace relational civility (WRC), positive relational management (PRM), and emotional intelligence (EI), as possible primary preventive resources to effectively deal with interpersonal mistreatment in the workplace (i.e., incivility). Since women endure workplace incivility more frequently than men, their well-being is particularly at risk. Thus, the possibilities for further research and primary prevention interventions in line with the achievement of the fifth Sustainable Development Goal (SDG 5) are discussed.

## Introduction

Uncivil behaviors are becoming more frequent in our post-modern society. In a 2002 survey of 2,000 American respondents, roughly four out of five considered disrespect, a lack of consideration, and rudeness serious issues, and three out of five believed that the situation was getting worse (Farkas and Johnson, 2002). The workplace is no exception. Due to globalization, rapid economic changes, and technological advancements, workers’ experience of the 21st century labor market could be stressful (Blustein et al., 2018), since coping with continuous change is often very demanding (Wanberg and Banas, 2000). This new work environment, characterized by the great number, complexity, and fragmentation of workplace relationships, may increase incivility (Pearson et al., 2000). Moreover, a work and information overload can lead to an increased perception of time pressures and thus induce workers to be less polite in their interpersonal behavior (Pearson et al., 2000; Pearson and Porath, 2005). Between 10 and 20% of workers reported witnessing incivility daily, while 20–50% affirmed that they had been the direct target of mistreatment in their workplace (Griffin and O’Leary-Kelly, 2004; Pearson and Porath, 2005). Notably, women endure workplace incivility more frequently than men (Cortina et al., 2001). In order to achieve gender equity, as defined by the fifth Sustainable Development Goal (United Nations, 2018) and promote well-being among women in the workplace, new theoretical and intervention approaches, such as intervention in the primary prevention framework (Hage et al., 2007; Kenny and Hage, 2009; Di Fabio 2017a), should be considered. This would help to confront incivility and create more civil workplace environments, from which all employees would likely benefit. The article discusses several constructs related to the primary prevention approach, based on advanced relational competencies, which would like to reduce the frequency of incivility (i.e., reducing risk), also as a mean to face gender inequality (Kalev and Deutsch, 2018) and shape healthier relational cultures (building strengths) advantageous for both women and men (Saxena et al., 2019).

## Consequences of Incivility

Workplace incivility is defined as low-intensity deviant behavior with ambiguous intent to harm the target (Andersson and Pearson, 1999). Uncivil behaviors are stressors that can lead to negative health consequences (e.g., depression, physical symptoms; Jex et al., 1992; Spector and Jex, 1998). On a psychological level, experiencing interpersonal mistreatment could harm one’s self-image (i.e., offense to self; Cornish-Bowden, 2004). Experiencing incivility can decrease an individual’s self-esteem (Frone, 2000), self-efficacy (Mikkelsen and Einarsen, 2002), self-confidence (Vartia, 2001), and well-being (Lapierre et al., 2005). Empirical evidence suggests that incivility is negatively associated with job satisfaction, psychological well-being, and life satisfaction. Moreover, its occurrence is connected with higher levels of job stress, job withdrawal, and psychological distress (Lim and Cortina, 2005). Interestingly, for women, the negative relationship between incivility and overall job satisfaction is stronger than the relationship between sexual aggression and overall job satisfaction (Lapierre et al., 2005). Thus, the occurrence of workplace incivility could be sufficient to determine a decrease in women’s occupational, psychological, and physical health (Lapierre et al., 2005; Lim and Cortina, 2005).

## From Workplace Incivility to Workplace Relational Civility

The contemporary prevention approach (Hage et al., 2007; Kenny and Hage, 2009) is focused on both reducing risks and building strengths among individuals (e.g., promoting individual resources; Di Fabio and Saklofske, 2014) and within organizations (Tetrick and Peiró, 2012; Di Fabio, 2017b). Traditionally, the work and organizational literature has focused on workplace incivility rather than civility in the workplace (Andersson and Pearson, 1999; Cortina et al., 2001; Pearson et al., 2001; Schilpzand et al., 2016). Nevertheless, to establish the optimal conditions for developing adaptive relationships among co-workers and thus promote well-being in the workplace, civility is mandatory (Blustein, 2011). Civility implies respect, courtesy, and awareness of the rights of others (Carter, 1998; Maree, 2012), and it is intrinsically relational (Di Fabio and Gori, 2016).

Workplace relational civility (WRC) has been defined as a relational style characterized by respect and concern for both the self and others, interpersonal sensitivity, personal education, and kindness toward others (Di Fabio and Gori, 2016), and it is described by three dimensions: (1) *relational decency*, (2) *relational culture*, and (3) *relational readiness*. Relational decency implies the ability to understand the relational dynamics of a given situation and constructively contribute to the relationships within the workplace. Relational culture refers to the culture’s influence in shaping kind and polite relationships among people. Relational readiness concerns the ability to quickly understand others’ feelings and show proactive sensibility. The relationships between WRC and the outcomes of workplace incivility have been empirically tested (Di Fabio et al., 2016; Di Fabio and Gori, 2016). The WRC was showed to be associated with higher levels of self-esteem and perceived social support. Perceived social support refers to the degree with which family, friends, and significant others are experienced as supportive and available. The association with perceived social support is particularly interesting for secondary (i.e., when the first symptoms are emerging) and tertiary prevention interventions (i.e., reducing the impact of an already-established problem; Caplan, 1964), since social support can buffer the detrimental effects of an unsafe workplace climate (van Emmerik et al., 2007). WRC is also related to both hedonic and eudaimonic well-being (Di Fabio et al., 2016; Di Fabio and Gori, 2016). Hedonic well-being consists of a cognitive evaluation component (i.e., satisfaction with life; Diener et al., 1985) and an affective evaluation component (i.e., the prevalence of positive emotions over negative emotions; Watson et al., 1988). By contrast, eudaimonic well-being is described as an individual’s optimal functioning and self-realization (i.e., meaning in life; Vázquez et al., 2006; Ryff and Singer, 2008).

## Positive Relational Management

Relationships are fundamental for people’s well-being (Rigby, 2000; Gallagher and Vella-Brodrick, 2008; Suldo et al., 2009; Ferguson and Goodwin, 2010) and within organizations (Tetrick and Peiró, 2012). The ability to dialectically integrate work and relationships, strengthening the aspects of the self in a relational environment, is a central aspect of the Positive Self and Relational Management model (Di Fabio and Kenny, 2016). Positive relational management (PRM) refers to an individual’s resources that are useful for relational adaptation within the workplace and beyond, and it is described by three dimensions (Di Fabio, 2016), namely, (1) *respect* (i.e., my respect for others, the respect of others for me, and my respect for myself), (2) *caring* (i.e., my care for others, the care of others for me, and my care for myself), and (3) *connectedness* (i.e., my connectedness with family members, friends, significant others, and reciprocity). PRM is associated with perceived social support (Pearson’s *r* ranging between 0.41 and 0.46; (Di Fabio, 2016). Thus, PRM resources appear useful for building positive and supportive relationships within the workplace. PRM also showed a strong connection with hedonic well-being (Pearson’s *r* ranging between 0.49 and 0.52; Di Fabio, 2016). Those who were more able in PRM also experienced higher satisfaction with their own life. Finally, PRM was empirically studied in reference to aspects of eudaimonic well-being (Di Fabio, 2016). The PRM scores were positively correlated with individuals perceiving their life as meaningful (Pearson’s *r* ranging between 0.39 and 0.57) and flourishing (Pearson’s *r* ranging between 0.41 and 0.68; Di Fabio, 2016; Di Fabio and Kenny, 2019). “Flourishing” encompasses purpose in life, positive relationships, engagement, competence, self-esteem, optimism, and contribution toward the well-being of others (Diener et al., 2010; Seligman, 2012; Huppert and So, 2013). Thus, PRM resources could not only increase well-being on an individual level but also potentially contribute to general workplace well-being.

## Emotional Intelligence and Emotional Intelligence Competencies

Emotional intelligence (EI) has been defined as the ability to discriminate and express emotions, assimilate emotions in thoughts, and regulate emotions in the self and others (Mayer et al., 2000b). EI is described by three categories of abilities: (1) appraisal and expression of emotions, (2) regulation of emotions, and (3) using emotions for solving problems (Salovey and Mayer, 1990). Although the literature agrees on the definition of EI, several different models have been proposed (Boyatzis, 2009; Cherniss, 2010). Historically, a first distinction has been made between ability-based EI, which refers strictly to the cognitive abilities required in the processing and use of emotional information, and mixed models which instead incorporate a wide range of personality variables (Petrides and Furnham, 2000; Mayer et al., 2000a). Subsequently, several scholars (Saklofske et al., 2003; Ashkanasy and Daus, 2005; Stough et al., 2009) have distinguished two principal EI models: ability-based models (Mayer et al., 2000a) and trait EI models, which encompass self-reported EI (Bar-On, 2004) and trait emotional self-efficacy measures (Petrides and Furnham, 2000, 2001, 2003). Another possible distinction around EI has emerged (Cherniss, 2010). Models that refer to the basic abilities of emotion recognition, reasoning, and regulation are categorized as EI models (Mayer et al., 2000a), whereas models that imply personal qualities that contribute to positive work-related performance (Boyatzis et al., 2000; Petrides and Furnham, 2000; Mayer et al., 2000a) are considered models of emotional intelligence competencies (EIC). Recently a holistic view of EI, which include multiple levels, has been proposed (Boyatzis, 2018). According to the multi-level theory framework, EI is articulated on three levels: basic ability/trait, self-perceived level, and behavioral level.

Despite the fragmented framework around EI and EIC, the empirical evidence and implication of these constructs on well-being appear to be clear. The higher scores on the self-reported measures of EI (i.e., EQ-i, Trait Emotional Intelligence Questionnaire) were associated with greater resilience and a greater sense of life satisfaction (Di Fabio and Saklofske, 2014). This result suggested that intervene on people’s perceptions of their emotional abilities can contribute potentially to their hedonic well-being. On the basis of this study, eudaimonic well-being has also been addressed in terms of its relationship with EI (Di Fabio and Kenny, 2019.). The trait EI scores appeared to be strongly related to the individual’s perception of a meaningful life (Di Fabio and Kenny, 2019) and flourishing (Di Fabio and Kenny, 2019). By contrast, ability-based EI appeared to poorly contribute to both hedonic and eudaimonic well-being (Bhullar et al., 2013). Nevertheless, ability-based EI (Mayer Salovey Caruso Emotional Intelligence Test, MSCEIT; Mayer et al., 2002) is associated with an increased perceived social support. In other words, people who reported a greater ability in perceiving, understanding, and managing emotions and using them to facilitate thought also perceived more social support (Di Fabio, 2015).

In terms of contributing to problem-solving, social responsibility, and impulse control, EI is showed to be connected to how people manage conflict in the workplace (Hopkins and Yonker, 2015). A recent study explored the connection between a wide pool of EI instruments (i.e., MSCEIT, EQ-i, Trait Emotional Intelligence Questionnaire) and individuals’ resilience and hedonic well-being (i.e., satisfaction with life; Di Fabio and Saklofske, 2014).

## Conclusion

Incivility is a serious threat to people’s well-being (Lapierre et al., 2005; Lim and Cortina, 2005). Women are particularly vulnerable to the detrimental effects of workplace aggression, since they experience it more frequently (Cortina et al., 2001). Thus, promoting well-being in the workplace and preventing certain unsafe dynamics from establishing themselves could be considered a promising strategy to reach gender equity (United Nations, 2018) as well as to advance women’s careers within organizations (Hopkins and Bilimoria, 2008; O’Neil et al., 2008).

Identical working conditions can generate a gap between women and men in terms of well-being and job opportunities since unhealthy relational work environments particularly penalize women. For instance, women are more likely to experience psychological distress due to incivility (Abubakar, 2018) and this could hinder an equal career development across gender (e.g., women have a higher risk for long-term sickness absence than men; Lidwall and Marklund, 2006). Moreover, incivility could be used as a way to demonstrate power and thus prescribe the “appropriate” gender behavior among non-conforming women and men, which usually underpins gender inequality (Kalev and Deutsch, 2018).

The primary prevention approach (Kenny and Hage, 2009; Di Fabio, 2017a) and the psychology of sustainability and sustainable development (Di Fabio, 2017b; Di Fabio and Rosen, 2018) focus on constructs that are potentially affected by interventions. In this sense, WRC, PRM, EI, and EIC, as with every resource that is conceived as trainable interpersonal and emotional abilities and skills, are worth taking into consideration (Slaski and Cartwright, 2003; Leiter et al., 2011; Cherry et al., 2012). All the aforementioned constructs appeared to be related to social support, indicating that being able to build positive and supportive relationships in the workplace could hinder the occurrence of interpersonal mistreatment. Social support could be also able to buffer the detrimental outcomes related to incivility (Schilpzand et al., 2016) and stress in general (Väänänen et al., 2003; González-Morales et al., 2006; Peiró, 2008). Indeed, social support from supervisors and co-workers appeared to favor people’s job satisfaction (Acker, 2004). Nevertheless, social support did not automatically imply advanced relational competencies, which may contribute to shape and support a preventive, advanced, and competent relational culture of an organization. Promoting relational awareness, strengths, and resources in a primary prevention perspective could play a crucial role in avoiding the establishment of dangerous relational dynamics. Interestingly, EIC could influence the way people manage conflict in the workplace (Hopkins and Yonker, 2015) and thus prevent the emergence of unsafe interpersonal conditions. PRM also could enhance individuals’ relational strengths and improve workers’ quality of life. Overall, building early and preventively people’s advanced awareness and relational competencies can contribute to shaping an adaptive relational culture within organizations, which is important for fostering women’s meaning of work (Grossman and Chester, 1989; Thory, 2016) and wellbeing (Zurbrügg and Miner, 2016). Interestingly, acting on these constructs may be relevant for women since women are more likely to be victimized, but may benefit all the workers. Indeed, a healthy relational environment affects all workers (Nielsen et al., 2017).

In general, both hedonic and eudaimonic well-being appear to be affected by WRC, PRM, EI, and EIC. However, some conflicting evidence has emerged from the literature analysis in relation to ability-based EI and hedonic well-being (Bhullar et al., 2013; Di Fabio and Saklofske, 2014). Overall, the contribution of ability-based EI to individuals’ satisfaction with life appeared modest, if not absent. Instead, the evidence regarding the relationship between WRC, PRM, EIC, and well-being seems more robust (Di Fabio and Saklofske, 2014; Di Fabio, 2016; Di Fabio et al., 2016; Di Fabio and Gori, 2016). Nevertheless, WRC and PRM are very novel constructs (Di Fabio, 2016; Di Fabio and Gori, 2016). Thus, further research should look to assess how they change over time by means of longitudinal studies. Moreover, the degree of WRC and PRM interventions’ effectiveness regarding well-being and workplace incivility should be assessed to offer evidence of causality and indication about the optimal and most efficient intervention duration. Cultural and ethnic background effects should be assessed as well. The Psychology of Harmony and Harmonization (Di Fabio and Tsuda, 2018) highlighted that the value of balancing process related to individuals’ relationality aspects (inner relationality, relationality with others, relationality with contexts in a temporal and geographical perspective) might be similar across cultures. However, the optimal level of balance between those aspects could be different between cultures (Sharma, 2012). In such sense, more research should be carried on to define which aspects encompassed by the primary prevention constructs presented in this study are more suitable for intervention in different regions of the world.

Future research has to take in consideration also other contextual and temporal aspects of this perspective, as, for example, type of organization and setting, gender and age mix of people, and how long must these relational competencies be practiced in the organization to see any type of measurable result.

Finally, in terms of limitations, since the literature showed improvement mainly on the experience of individuals, group level measures are needed to investigate on multiple levels (e.g., group, organization) the outcomes of primary prevention interventions based on the enhancement of relational competencies.

In conclusion, it seems that the primary prevention approach (Hage et al., 2007; Kenny and Hage, 2009; Di Fabio, 2017a) could effectively contribute to gender equity by promoting well-being in an environment in which the recent changes due to globalization and technological advancements (Savickas, 2011; Blustein et al., 2018) are making incivility more frequent (Farkas and Johnson, 2002), especially toward women (Cortina et al., 2001).

## Data Availability

No datasets were generated or analyzed for this study.

## Author Contributions

AD and MD ideated the structure, analyzed the literature, and wrote the manuscript.

### Conflict of Interest Statement

The authors declare that the research was conducted in the absence of any commercial or financial relationships that could be construed as a potential conflict of interest.
